# Supplementary material for: Multifunctional TiO2 Nanotube-Matrix Composites with Enhanced Photocatalysis and Lithium-Ion Storage Performances
Source: Materials (Basel). 2023 Mar 29;16(7):2716. doi: 10.3390/ma16072716 (PMC10096361; doi:10.3390/ma16072716)
Supplement: Supplementary file 1 [file materials-16-02716-s001.zip › materials-2276410-supplementary.pdf]

Supplementary Material

Multifunctional TiO_2 Nanotube-Matrix Composites with Enhanced Photocatalysis and Lithium-Ion Storage Performances

Mengmeng Zhang, Hui Li * and Chunrui Wang *

Shanghai Institute of Intelligent Electronics and Systems, College of Science, Donghua University, Shanghai 201620, China
* Correspondence: huili@dhu.edu.cn (H.L.); crwang@dhu.edu.cn (C.W.)

Material Characterization

The morphology of as-prepared samples was detected by the field emission scanning electron microscope (FESEM, Hitachi, S-4800) and the high-resolution transmission electron microscopy (HRTEM, TECHAI G2S-TWIN). The structures were analyzed with X-ray diffraction (XRD, Rigaku-D/max-2550VB+/PC X-ray diffractometer, λ=1.54056 Å). Various samples' Raman spectra were recorded on a LABRAM-1 Bconfocal laser micro-Raman spectrometer (532 nm radiation) at room temperature.

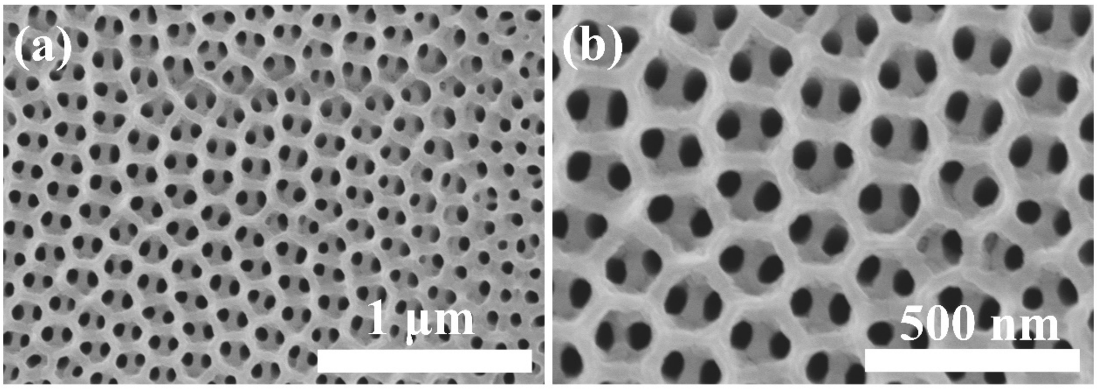

Figure S1. (a) Low magnification and (b) high magnification SEM images of as-annealed secondary oxidized TiO_2 nanotube (TiO_2 NT).

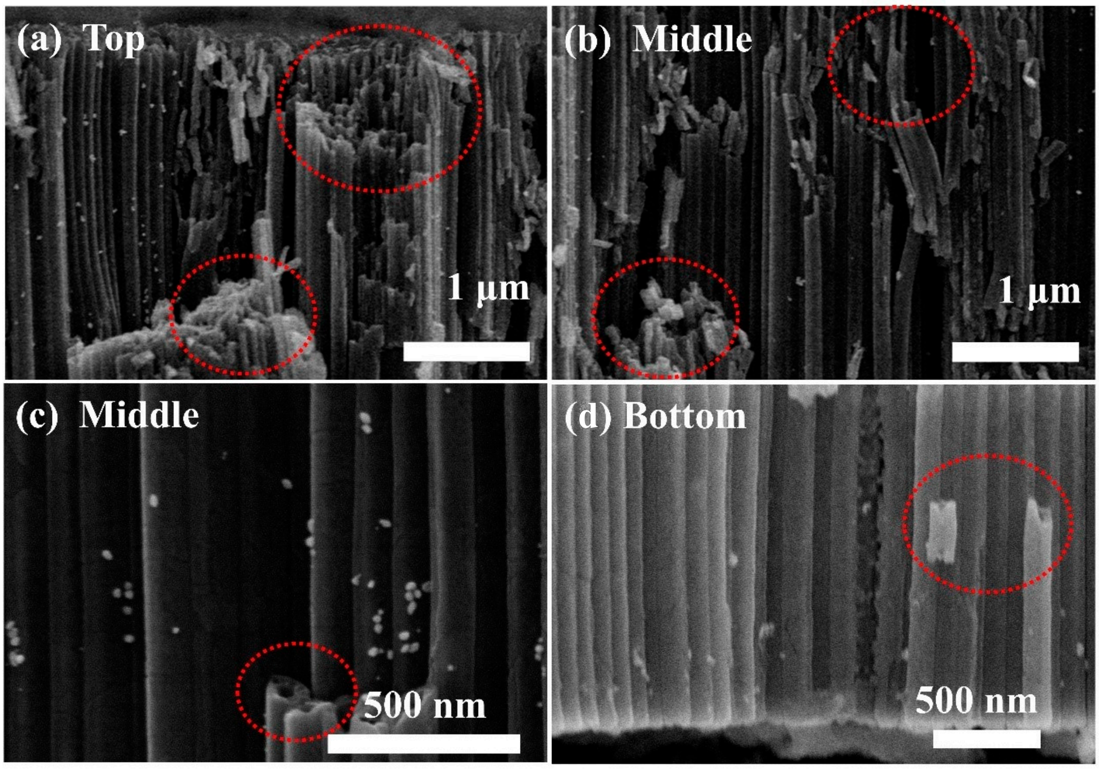

Figure S2. SEM images of the (a) top, (b,c) middle, and (d) bottom parts of the TiO_2 NT@Au NP composite.

SEM images of different parts of the TiO_2 NT@Au NP composite show that there are no gold nanoparticles attached to the inner surface of TiO_2 nanotubes (as marked positions). Thus, Au nanoparticles only adhere to the outer surface of the TiO_2 nanotubes. And The adhesion of Au NP to TiO_2 NT may be related to the defects of the TiO_2 nanotubes, which are distributed on the top and outer surfaces of the nanotubes [1].

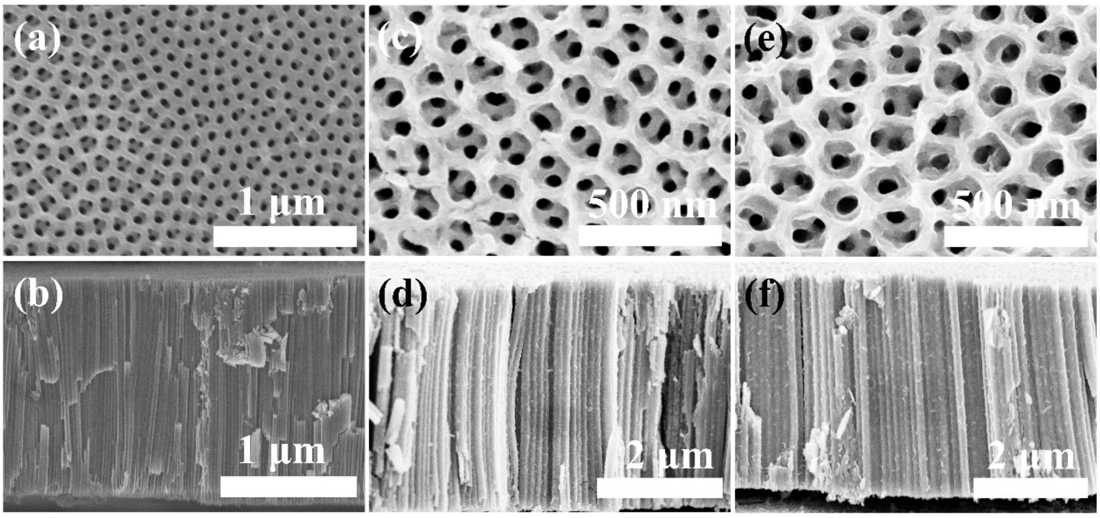

Figure S3. When the surfactant (PVP) is not present, the MoS_2 nanosheet cannot be coated on the TiO_2 nanotube, and (a,b) is the corresponding SEM images. SEM images of TiO_2 nanotube@MoS_2 nanosheet composites when the mass of PVP surfactant is (c,d) 0.1 g (TiO_2 NT@MoS_2 NS, 0.1 g) and (e,f) 0.3 g (TiO_2 NT@MoS_2 NS, 0.3 g).

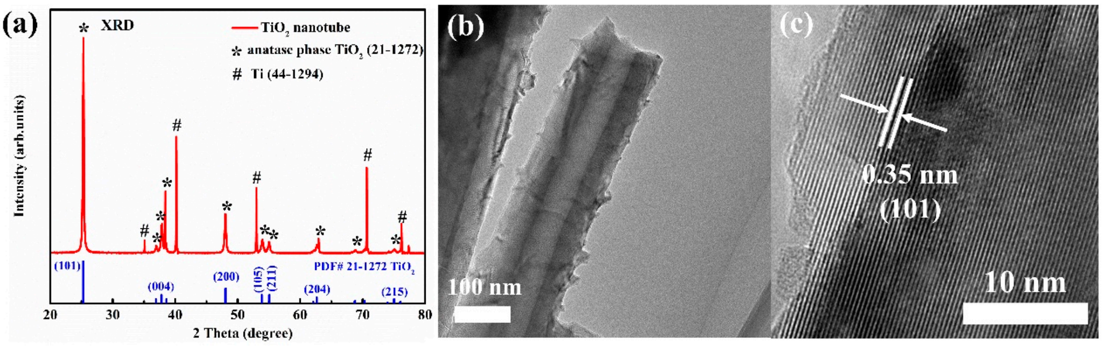

Figure S4. (a) XRD of TiO_2 nanotube, as well as (b) TEM and (c) HRTEM images of TiO_2 nanotube.

The XRD pattern of TiO_2 NT (Figure S3a) shows the characteristic peaks of anatase phase TiO_2 (PDF #21-1272) and Ti (PDF #44-1294) substrate without impurity peaks, revealing the anatase crystal structure of the TiO_2 NT [2–4]. High-resolution TEM (HRTEM) images of TiO_2 NTs (Figure S3c) display a typical lattice spacing of 0.35 nm, which is ascribed to the (101) plane of anatase phase TiO_2 [4–6].

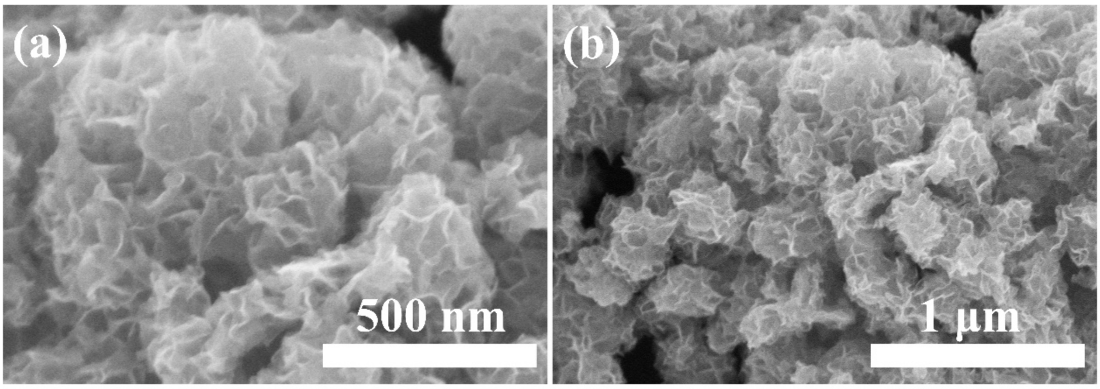

Figure S5. (a) High magnification and (b) low magnification SEM images of MoS_2 powder.

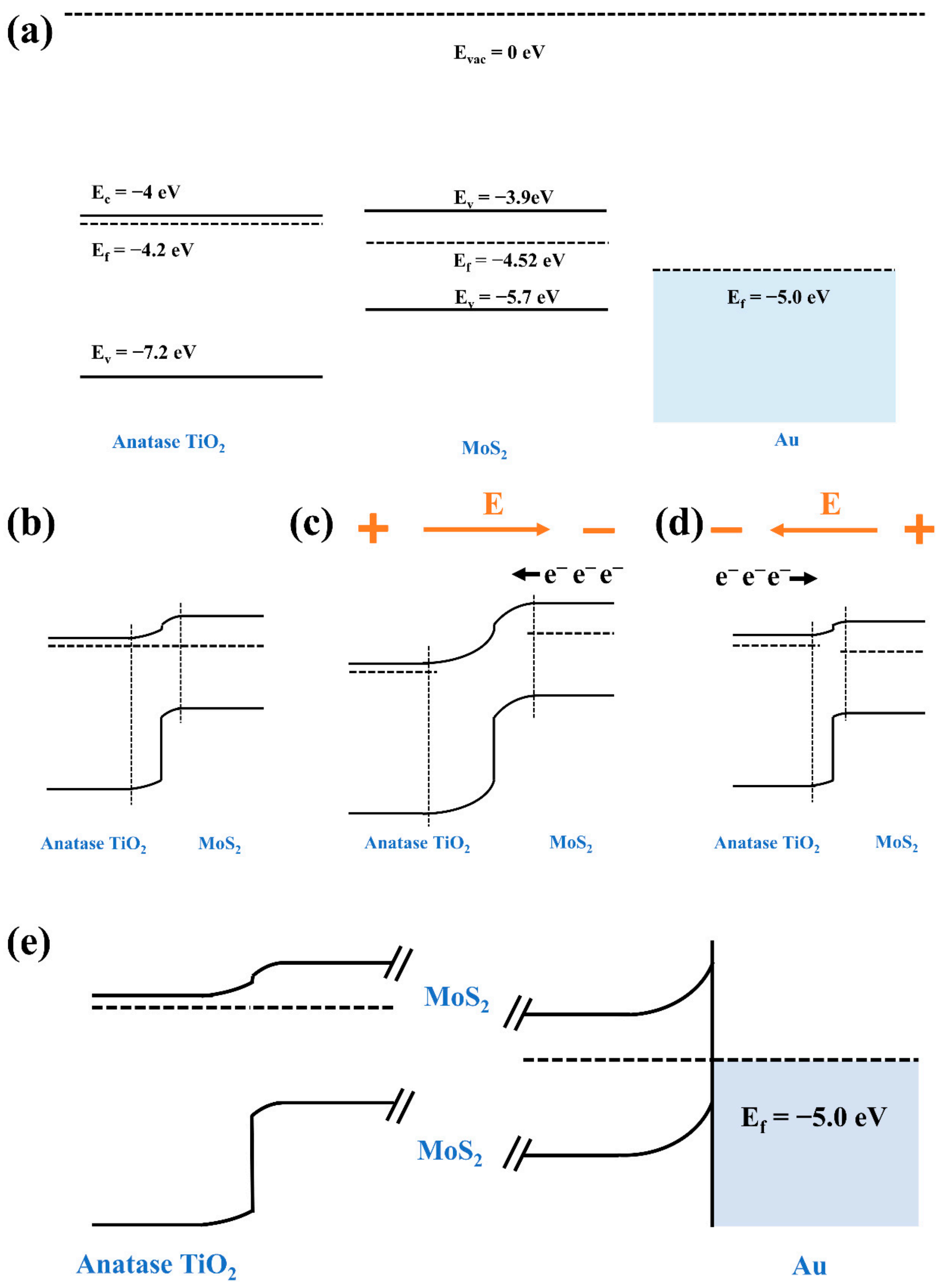

Figure S6. (a) Energy-band diagrams of TiO_2 nanotube, MoS_2 nanosheet and Au nanoparticle. (b–d) Energy-band diagrams of TiO_2 nanotube@MoS_2 nanosheet composite. (e) Energy-band diagrams of TiO_2 nanotube@MoS_2 nanosheet@ Au nanoparticle composite.

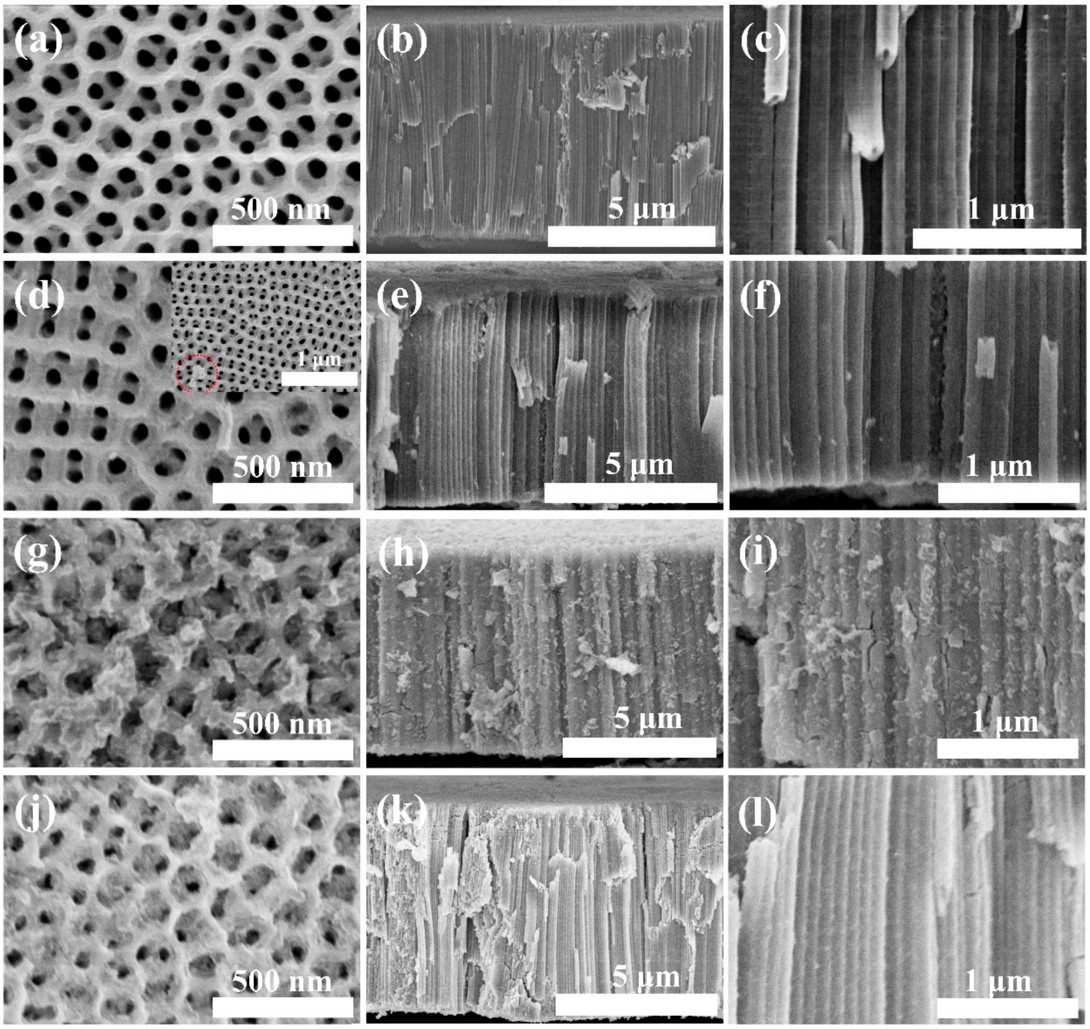

Figure S7. SEM images of four different anode materials, after 50 cycles, at 100 μA cm^{-2}. SEM images of cycled (a–c) TiO_2 nanotube anode, (d–f) TiO_2 nanotube@Au nanoparticle anode, (g–i) TiO_2 nanotube@MoS_2 nanosheet anode and (j–l) TiO_2 nanotube@MoS_2 nanosheet@ Au nanoparticle anode.

References

1. Zhang, M.M.; Lu, A.J.; Li, H.; Li, M.; Wang, J.L.; Wang, C.R. Defective TiO_2-Supported Dual-Schottky Heterostructure Boosts Fast Reaction Kinetics for High Performance Lithium-Ion Storage. *ACS Appl. Energy Mater.* **2023**, doi:10.1021/acsaem.2c03668.
2. Chen, K.; Guo, H.N.; Li, W.Q.; Wang, Y.J. MOF-derived Core-Shell CoP@NC@TiO_2 Composite as a High-Performance Anode Material for Li-ion Batteries. *Chem.-Asian J.* **2021**, 16, 322–328.
3. Liu, Y.; Luo, Y.F.; Elzatahry, A.A.; Luo, W.; Che, R.C.; Fan, J.W.; Lan, K.; Al-Enizi, A.M.; Sun, Z.K.; Li, B.; et al. Mesoporous TiO_2 Mesocrystals: Remarkable Defects-Induced Crystallite-Interface Reactivity and Their in Situ Conversion to Single Crystals. *ACS Central Sci.* **2015**, 1, 400–408.
4. Tan, B.Y.; Zhang, X.H.; Li, Y.J.; Chen, H.; Ye, X.Z.; Wang, Y.; Ye, J.F. Anatase TiO_2 Mesocrystals: Green Synthesis, In Situ Conversion to Porous Single Crystals, and Self-Doping Ti^{3+} for Enhanced Visible Light Driven Photocatalytic Removal of NO. *Chem.-Eur. J.* **2017**, 23, 5478–5487.
5. Liao, J.Y.; Lei, B.X.; Wang, Y.F.; Liu, J.M.; Su, C.Y.; Kuang, D.B. Hydrothermal Fabrication of Quasi-One-Dimensional Single-Crystalline Anatase TiO_2 Nanostructures on FTO Glass and Their Applications in Dye-Sensitized Solar Cells. *Chem.-Eur. J.* **2011**, 17, 1352–1357.
6. Fang, Y.Z.; Zhang, Y.Y.; Miao, C.X.; Zhu, K.; Chen, Y.; Du, F.; Yin, J.L.; Ye, K.; Cheng, K.; Yan, J.; et al. MXene-Derived Defect-Rich TiO_2@rGO as High-Rate Anodes for Full Na Ion Batteries and Capacitors. *Nano-Micro Lett.* **2020**, 12, 128.
